# Medical Department of the University of Louisville

**Published:** 1866-08

**Authors:** 


### MEDICAL DEPARTMENT OF THE UNIVERSITY OF LOUISVILLE.

We have received a pamphlet showing the re-organization of the Faculty of the above-named institution, and find that two of the old Faculty resigned the first of this year, and that the other members were removed by the Board of Trustees.

"The Record of Facts in relation to the Dismissal of the Faculty," as given in the pamphlet before us, was written by the old members, and goes to show that undue *pressure* was brought to bear upon the City Council, whose duty it was to fill several vacancies in the Board of Trustees which occurred about the first of the present year, from expiration of their term of service. It is alleged that the pressure was exerted by the aspirants for places in the Faculty, and, as is hinted, political reasons made the basis of operations.

If this be so, we think it augurs badly for the future prospects of this old and respectable institution. If proscription is to be the order of the day, on *neutral* or middle ground, with medical colleges, and the proscribers savor of radicalism, it strikes us that the field for supply of students will be somewhat limited to such schools. It is not at all probable that students, convenient to Western and Northern colleges, will come down to Louisville, nor is it likely that students from a warmer climate would feel comfortable in Louisville during winter under such circumstances as are said to surround the new Faculty. Dr. Bemiss, a member of the old Faculty, received and accepted an invitation to a chair in the Medical Department of the University of Louisiana, New Orleans.

